# C57BL/KsJ-*db/db*-*Apc^Min/+^* Mice Exhibit an Increased Incidence of Intestinal Neoplasms

**DOI:** 10.3390/ijms12118133

**Published:** 2011-11-18

**Authors:** Kazuya Hata, Masaya Kubota, Masahito Shimizu, Hisataka Moriwaki, Toshiya Kuno, Takuji Tanaka, Akira Hara, Yoshinobu Hirose

**Affiliations:** 1 Department of Tumor Pathology, Gifu University Graduate School of Medicine, 1–1 Yanagido, Gifu 501–1194, Japan; E-Mails: tkuno@gifu-u.ac.jp (T.K.); ahara@gifu-u.ac.jp (A.H.); yhirose@gifu-u.ac.jp (Y.H.); 2 Kamiishidu Division, Sunplanet Co., Gifu 503–1602, Japan; 3 Department of Medicine, Gifu University Graduate School of Medicine, Gifu 501–1194, Japan; E-Mails: samurai0201@yahoo.co.jp (M.K.); shimim-gif@umin.ac.jp (M.S.); hmori@gifu-u.ac.jp (H.M.); 4 Department of Oncologic Pathology, Kanazawa Medical University, Ishikawa 920–0293, Japan; 5 Cancer Research and Prevention (TCI-CaRP), Tohkai Cytopathology Institute, Gify 500–8285, Japan

**Keywords:** C57BL/KsJ-db/db, C57BL/6J-Apc^*Min*/+^, Type 2 diabetes mellitus, colon carcinogenesis, animal model

## Abstract

The numbers of obese people and diabetic patients are ever increasing. Obesity and diabetes are high-risk conditions for chronic diseases, including certain types of cancer, such as colorectal cancer (CRC). The aim of this study was to develop a novel animal model in order to clarify the pathobiology of CRC development in obese and diabetic patients. We developed an animal model of obesity and colorectal cancer by breeding the C57BL/KsJ-*db/db* (db/db) mouse, an animal model of obesity and type II diabetes, and the C57BL/6J-*Apc^Min/+^* (Min/+) mouse, a model of familial adenomatous polyposis. At 15 weeks of age, the N9 backcross generation of C57BL/KsJ-*db/db*-*Apc^Min/+^* (db/db-Min/+) mice developed an increased incidence and multiplicity of adenomas in the intestinal tract when compared to the db/m-Min/+ and m/m-Min/+ mice. Blood biochemical profile showed significant increases in insulin (8.3-fold to 11.7-fold), cholesterol (1.2-fold to 1.7-fold), and triglyceride (1.2-fold to 1.3-fold) in the db/db-Min/+ mice, when compared to those of the db/m-Min/+ and m/m-Min/+ mice. Increases (1.4-fold to 2.6-fold) in RNA levels of insulin-like growth factor (IGF)-1, IRF-1R, and IGF-2 were also observed in the db/db- Min/+ mice. These results suggested that the IGFs, as well as hyperlipidemia and hyperinsulinemia, promoted adenoma formation in the db/db-Min/+ mice. Our results thus suggested that the db/db-Min/+ mice should be invaluable for studies on the pathogenesis of CRC in obese and diabetes patients and the therapy and prevention of CRC in these patients.

## 1. Introduction

Epidemiological studies have shown that obesity and diabetes mellitus may be one of the risk factors for colorectal cancer (CRC) development [1,2]. To date the underlying mechanisms of how obesity and diabetes promote colon carcinogenesis remain unknown, although insulin-resistance and hyperinsulinemia are proposed to be responsible for the risk factor [3]. Excess body weight is a major determinant for the development of insulin resistance with associated hyperinsulinaemia and hyperglycemia, and further leads to CRC development [4]. Certainly, insulin resistance and hyperinsulinaemia are key biological mechanisms underlying the relationship between adiposity and tumor development [5]. Recently, the anti-diabetic drug, metformin, in addition to reduction of insulin resistance has shown anti-tumor properties [6], and is considered as a drug to prevent and treat obesity-related cancers, including CRC [7]. The pathophysiological and biological mechanisms underpinning the associations between excess body weight/obesity, type 2 diabetes, and CRC are proposed, and insulin resistance is at the heart of the matter [8]. However, there are several other candidate systems, including insulin-like growth factors (IGF), adipocytokines, and inflammatory cytokines [9]. One such hypotheses is the role of insulin-IGF axis, where chronic hyperinsulinemia is associated with decreased concentrations of IGF-binding protein1 (IGFBP-1) and IGFBP-2, leading to increased availability of IGF-I and concomitant changes in the cellular environment that favor tumor development. Indeed, inhibition of the activation of the IGF/IGF-1R axis resulted in suppression of colonic premalignant lesions in an obesity-associated colon cancer model, which was also associated with hyperlipidemia, hyperinsulinemia, and hyperleptinemia [10]. However, hyperinsulinemia is also associated with alterations in related molecular systems (sex steroid hormones and adipocytokines). In this context, novel researches using appropriate animal models are needed to investigate the detailed mechanisms. We previously reported that C57BL/KsJ-*db/db* mice with hyperleptinemia and hyperinsulinemia are highly susceptible to azoxymethane (AOM)-induced colon carcinogenesis. C57BL/KsJ-*db/db* mice received AOM developed high frequency of premalignant lesions [11,12], and the mice are useful for studies for identifying chemopreventive agents against obesity/diabetes-assosiated colon carcinogenesis [13,14]. Min/+ mice [15], known to develop a number of adenomas by the same mechanism or process as seen in humans, have frequently been used as an animal model of familial adenomatous polyposis (FAP) for investigation of carcinogenesis, prevention, and therapy of CRC [16–21].

In the current study, we aimed to develop a spontaneous animal model of obesity with adenoma formation in the intestinal tract in order to investigate obesity-associated events in obesity-associated intestinal tumorigenesis and prevent spontaneous intestinal tumor development in the model. The model was developed by breeding db/m mice with Min/+ mice and then backcrossing of db/m mice with the offsprings born from both strains of mice. The db/db-Min/+ mice obtained will give us the important implications for further exploration of the possible underlying events that affect the positive association between CRC and chronic diseases, obesity and diabetes. Our main goal was to assess the involvement of obesity-associated events, such as hyperinsulinemia, in intestinal carcinogenesis *in vivo*.

## 2. Materilas and Methods

### 2.1. Animals

C57BL/KsJ-*db*+/+m (db/m) and C57BL/6J-*Apc^Min/+^* (Min/+) mice were purchased from the Japan SLC, Inc. (Shizuoka, Japan) and from the Jackson Laboratory (Bar Harbor, ME), respectively. They were bred and genotyped in our facility for the project. In brief, after the db/m females were mated with a db/m-Min/+ male, we finally obtained three mouse strains, the db/db-Min/+, the db/m-Min/+, and the m/m-Min/+ mice at the N9 backcross generation by backcrossing db/m mice with the offsprings born from the mating between the db/m mice and the Min/+ mice (Figure S1), The *Apc* gene was detected by polymerase chain reaction (PCR), while the db/m gene was recognized by the body shape and coat color of mice.

Mice used for the study were maintained in the well-controlled room with a high-efficiency particulate air (HEPA) filter, a 12 h lighting (7:00–19:00), 25 ± 2 °C room temperature, and 55 ± 15% humidity. Mice (3–6 mice/cage) were housed in polycarbonate cages measuring W225 × D338 × H140 mm (Japan CLEA, Inc., Tokyo, Japan) with the floor covered with a sheet of roll paper (Japan SLC, Hamamatsu City, Japan). MF (Oriental Yeast Co., Ltd., Tokyo, Japan) was used as a basal diet throughout the study. Groundwater that was chlorine-treated and subjected to ultraviolet disinfection was used as drinking water in a bottle. We fully complied with the “Guidelines Concerning Experimental Animals” issued by the Japanese Association for Laboratory Animal Science and exercised due consideration so as not to cause any ethical problem.

### 2.2. Experimental Procedure

After weaning at 4 weeks of age, a total of 94 mice aged 5 weeks were used in the study. They included 18 males and 13 females of db/db-Min/+ mice, 23 males and 19 females of db/m-Min/+ mice and 11 males and 10 females of m/m-Min/+ mice, at 15 weeks of age, the mice were weighed and measured their lengths for BMI calculation, and thereafter underwent blood withdrawal from abdominal aorta under anesthetic and intestinal evisceration for counting intestinal adenomas.

### 2.3. Pathological and Immunohistochemical Analyses

In addition to the macroscopic examination, the volume of colonic tumors was determined. The mean volumes (mm^3^) of colonic tumors were calculated by the formula, (a) × (b) × (b)/2 where (a) and (b) were major axis and minor axes, respectively. After careful macroscopic observation, the small and large intestines were fixed in 10% buffered formalin and paraffin-embedded tissue blocks of whole intestine were made. These tissues were subjected to hematoxylin and eosin (H & E) staining for histopathology. In addition, immunohistochemistry of β-catenin and IGF-1R were performed using the labeled streptavidin-biotin method (LSAB kit; DAKO, Glostrup, Denmark), as previously described. Anti-β-catenin antibody (1:1,000 final dilution) obtained from Transduction Laboratories (catalog no. 610154; San Jose, CA, USA) and anti-IGF-1R antibody (1:100 final dilution) purchased from Santa Cruz Biotechnology, Inc. (sc-7907; Santa Cruz, CA, USA) were applied as primary antibodies. Negative control sections were immunostained without the primary antibodies.

### 2.4. Blood Chemistry

At 15 weeks of age, blood samples (0.5–1.0 mL/mouse) were collected for determination of total cholesterol, triglyceride and glucose by a simple measurement device (DRICHEM Fujifilm Medical Co., Ltd., Tokyo, Japan). The concentration of blood insulin was measured by the LBIS insulin measuring kit for mice (Shibayagi Co., Ltd., Gunma, Japan).

### 2.5. RNA Extraction and Quantitative Real-Time RT-PCR Analysis

A quantitative real-time RT-PCR analysis was carried. Total RNA was isolated from the scraped colonic mucosa of the male mice (*n* = 6 from each strain) using the RNAqueous-4PCR kit (Ambion Applied Biosystems, Austin, TX, USA). The cDNA was synthesized from 0.2 μg total RNA using the SuperScript III First-Strand Synthesis System (Invitrogen, Carlsbad, CA, USA). The primers used for the amplification of IGF-1, IGF-2, and IGF-1R specific genes were as follows: IGF-1 forward, 5-CTGGACCAGAGACCCTTTGC-3 and reverse, 5-GGACGGGGACTTCTGAGTCTT-3; IGF-2 forward, 5-GTGCTGCATCGCTGCTTAC-3 and reverse, 5-ACGTCCCTCTCGGACTTGG-3; and IGF-1R forward, 5-GTGGGGGCTCGTGTTTCTC-3 and reverse, 5-GATCACCGTGCAGTTTTCCA-3. Real-time PCR was done in a LightCycler (Roche Diagnostics Co., Indianapolis, IN, USA) with SYBR Premix Ex Taq (TaKaRa Bio, Shiga, Japan). The expression levels of the IGF-1, IGF-2, and IGF-1R genes were normalized to the β-actin gene expression level.

### 2.6. Statistical Analysis

Measurements are expressed as the mean value ± standard deviation (Mean ± SD), and differences if present were compared by one-way analysis of ANOVA and Tukey-Kramer’s multiple comparison’s test. The incidences of intestinal tumors were compared by Fisher’s exact probability test. The results were considered statistically significant if the *p* values were <0.05.

## 3. Results

### 3.1. General Observations

**Figure 1:**
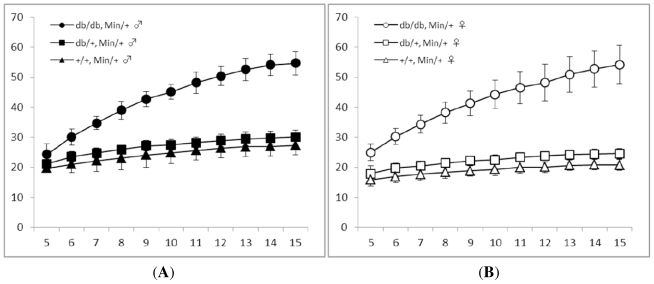
Body weight gains during the study: (**A**) males and (**B**) females.

As shown in Figure 1, the mean body weights of the db/db-Min/+ mice, either males (Figure 1(A)) or females (Figure 1(B)), were significantly heavier even at the age of 5 weeks than that of the db/m-Min/+ and m/m-Min/+ mice. The differences continued up to 15 weeks of age. The mean body weights of males and females of db/m-Min/+ mice were significantly higher than that of the m/m-Min/+ mice in either sex at 5 weeks of age, and this trend continued until 15 weeks of age. Table 1 summarizes the body length, body weight, and body mass index (BMI) at 15 weeks of age. The BMIs of the db/db-Min/+ mice (0.62 ± 0.04 in males and 0.63 ± 0.08 in females, *p* < 0.001 for each comparison) were significantly greater than those of the db/m-Min/+ (0.35 ± 0.03 in males and 0.30 ± 0.03 in females) and m/m-Min/+ mice (0.34 ± 0.05 in males and 0.27 ± 0.03 in females).

### 3.2. Serum Levels of Glucose, Total Cholesterol, Triglyceride and Insulin in Experimental Mice

The blood concentrations of glucose, total cholesterol, triglyceride, and insulin in the db/db-Min/+, db/m-Min/+, and m/m-Min/+ at 15 weeks of age are listed in Table 2. The measures of the db/db-Min/+ mice were higher than that of the db/m-Min/+ or m/m-Min/+ mice (0.001 < *p* < 0.05). However, the values of the db/m-Min/+ and m/m-Min/+ mice of either sex were comparable.

### 3.3. Tumors in the Intestinal Tract

**Figure 2:**
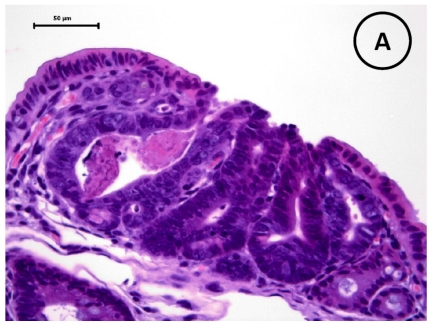

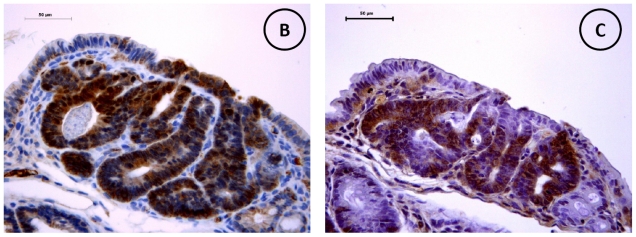
Histopathology of a colonic tumor developed in db/db-Min/+ mice. The tumor is histopathologically diagnosed as a tubular adenoma. Immunohistochemistry of β-catenin and IGF-1R in the tumor shows positive reaction of β-catenin in their cell membrane and cytoplasm and IGF-1R in their cytoplasm. Bars = 50 μm. (**A**) H & E stain; (**B**) β-catenin immunohistochemistry; and (**C**) IGF-1R immunohistochemistry.

Intestinal nodular tumors were observed in three strains of mice of each sex. As shown in Table 3, the total numbers of tumors, histopathologically tubular adenomas (Figure 2(A)), in small and large intestine of the db/db-Min/+ mice (60.5 ± 14.6 in males and 57.8 ± 12.7 in females, *p* < 0.001 for each comparison) were significant larger than the db/m-Min/+ mice (32.1 ± 7.5 in males and 32.6 ± 5.9 in females) and m/m-Min/+ mice (30.5 ± 7.7 in males and 30.9 ± 6.3 in females). The values of the db/m-Min/+ mice and m/m-Min/+ mice were comparable. The mean numbers of small intestinal tumors of the db/db-Min/+ mice of both sexes at the age of 15 weeks were also significantly greater than that of the db/m-Min/+ and m/m-Min/+ mice (*p* < 0.001). The incidence and multiplicity of colonic adenomas of the male and female db/m-Min/+ mice were greater than those of the db/m-Min/+ and m/m-Min/+ mice, and significant differences in the incidence were observed between the db/db-Min/+ and db/m-Min/+ (*p* < 0.005) or m/m-Min/+ males (*p* < 0.005), but not between the females. Histopathology of intestinal tumors developed in three strains of mice did not significantly differ. There were no significant differences in proliferation activities and apoptotic index in the tumor cells among three strains of mice (data not shown).

The mean volumes (mm^3^) of colon tumors were 11.95 ± 11.70 (n = 31) in the db/db-Min/+ mice, 13.63 ± 8.78 (n = 12) in the db/m-Min/+ mice, and 15.52 ± 13.67 (n = 10) in the m/m-Min/+ mice, and the values did not significantly differ among three strains of both sexes.

### 3.4. Expression Levels of IGF-1, IGF-2, and IGF-1R mRNAs

**Figure 3:**
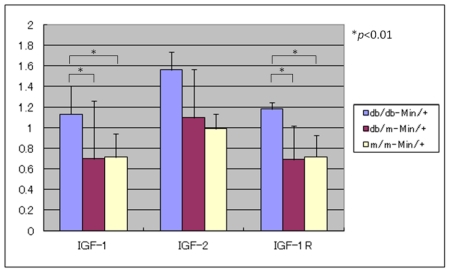
mRNA expression of IGF-1, IGF-2, and IGF-1R in the colonic mucosa of three strains of male mice (*n* = 6 from each strain).

The expression levels of IGF-1, IGF-2, and IGF-1R mRNAs in the colonic mucosa of the db/db-Min/+, db/m-Min/+ and m/m-Min/+ mice. As illustrated in Figure 3, the db/db-Min/+ mice showed significant increased mRNA levels of IGF-1, IRF-1R, and IGF-2 (1.4-fold to 2.6-fold increase), when compared to the db/m-Min/+ or m/m-Min/+ mice.

### 3.5. Immunohistochemistry of β-Catenin and IGF-IR in the Colonic Tumors

Immunohistochemical expression of β-catenin was accumulated in the nucleus and cytoplasm of the colonic adenoma cells (Figure 2(B)) developed in three strains of mice. IGF-IR was immunohistochemically expressed in the cytoplasm and cell membrane of the adenoma cells (Figure 2(C)) and the sustainability did not significantly differ among adenomas developed in three strains of mice. Also, adenomas developed in small intestine shows similar expression of β-catenin and IGF-1R (data not shown).

## 4. Discussion

In the current study, we generated three mouse strains, the db/db-Min/+, db/m-Min/+, and m/m-Min/+ mice, by breeding db/m mice with the Min/+ mice, and then backcrossing of the db/m mice with the offsprings born from both strains of mice. All three strains developed intestinal adenomas and the number of tumors of either small intestine or colon in the db/db-Min/+ mice was greater than that of the db/m-Min/+ and m/m-Min/+ mice. The db/db-Min/+ mice were heavier than db/m-Min/+ and m/m-Min/+ mice. As expected, our findings were in accordance with our previous report showing that C57BL/KsJ-db/db obese mice were highly susceptible to AOM-induced colon carcinogenesis. In addition, the db/db-Min/+ mice had hyperinsulinemia, diabetes, and hyperlipidemia. Unexpectedly, development of colonic tumors including adenocarcinoma was not remarkable, but the number was slightly greater than that (0.5–1.1 colon tumors/mouse) described in previous reports [22,23]. The findings suggest that induction of a number of colonic tumors including adenocarcinoma needs other either intrinsic or external stimuli [24]. mRNA levels of IGF-1, IRF-1R, and IGF-2 in the colonic mucosa of the db/db-Min/+ mice were greater than the db/m-Min/+ or m/m-Min/+ mice. Immunohistochemically, the expression of β-catenin was accumulated in the nucleus and cytoplasm of small and large intestinal adenoma cells, and IGF-1R in the cytoplasm. These findings suggest that up-regulation of the expression of IGFs and the receptor might play a role in intestinal tumorigenesis [25], and that hyperinsulinemia could affect receptor-mediated signaling [10,11,26–28] in the db/db-Min/+ mice.

There is accumulating evidence suggesting that hyperinsulinemia and hyperlipidemia are involved in colon carcinogenesis in obese and diabetic rodents [29,30]. Several epidemiological studies indicate that diabetic patients with hyperinsulinemia have increased risk for CRC [27,31–35]. An experimental animal study also showed that continuous injections of insulin promote AOM-induced colon carcinogenesis in rats [36]. Hence, it seems likely that hyperinsulinemia in the db/db-Min/+ mice enhanced the development of intestinal adenomas in the current study. Regarding the mode of action, IGF-1 pathway plays a role in insulin-related tumor promotion in the colon. IGF-1 binds to the IGF-1R, activates a signal cascade, and triggers cell proliferation in several tissues, including colon [10,27,31]. Because of the homology with the insulin receptor, insulin at supra-physiological levels also binds to and activates the IGF-1R [37]. Furthermore, hyperinsulinemia was shown to indirectly increase bioavailability of IGF-1 by regulating the expression levels of IGF-binding proteins [38,39]. Our findings that IGF-1R expression was immunohistochemically up-regulated in the intestinal adenomas were in accordance with other studies showing that the IGF-1R is overexpressed in human CRC. Accordingly, it is possible that hyperinsulinemia in the db/db-Min/+ mice activates the signaling cascades involving the IGF-1R, resulting in a proliferative response.

In this study, we noticed hypelipidemia in the db/db-Min/+ mice, which is also a risk factor of human CRC development [40–43]. Experimentally, hyperlipidemia was found in intestinal carcinogenesis models with obese [14] and Min/+ mice [44]. Improvement of hyperlipidemia by certain drugs or natural compounds resulted in reduction and prevention of intestinal carcinogenesis in these models [10,13,14,22,23,28,44–47]. Thus, hyperlipidemia also contributes to a high incidence of spontaneous intestinal tumors in the db/db-Min/+ mice.

In the current study, we observed that the congenic db/db-Min/+ mice increase intestinal tumors compared to either transgenic counterpart. However, the volume of intestinal tumors did not significantly differ among the db/db-Min/+, db/m-Min/+ and m/m-Min/+ mice. Since differences in the indices of proliferation and apoptosis in the tumors were not observed among the three strains of mice (data not shown), there may be other factors that affect the growth of intestinal tumors. Recently Endo *et al.* [48] reported that the growth of colorectal tumors is dramatically inhibited in the *db/db* mice. Therefore, the leptin signaling might be important for colorectal tumor growth.

In conclusion, our findings described here indicate that the db/db-Min/+ mice with hyperinsulinemia and hyperlipidemia developed many intestinal adenoma, and the number was much greater than the db/m-Min/+ and m/m-Min/+ mice. The results observed were considered to occur through hyperinsulinemia and modulation of insulin-IGF axis. Since several biological events other than hyperinsulinemia/hyperlipidemia are also reported to be involved in the association between obesity/excess body weight, insulin resistance, and CRC development, such as chronic inflammation (inflammatory cytokines), glucose toxicity, advanced glycation end products product metabolism, and adipocytokies, further studies will be necessary to reveal the specific determinants that are responsible for the correlation between obesity and/or diabetes and colon carcinogenesis. Our db/db-Min/+ mice could be used for such studies.

## Supplementary Material



## Figures and Tables

**Table 1:** Body weight, body length and BMI of mice at 15 weeks of age.

Gender	Genotype		No. of mice examined	Body weight (g)	Body length (cm)	BMI (g/cm^2^)

	db/db type	Apc^Min/+^ type				
Males	db/db	Min/+	18	54.7 ± 3.9 a,b	9.38 ± 0.30 c	0.62 ± 0.04 b
	db/m	Min/+	23	30.2 ± 2.3 d	9.23 ± 0.38	0.35 ± 0.03 e
	m/m	Min/+	11	27.3 ± 3.2 f	9.00 ± 0.30	0.34 ± 0.05 f

Females	db/db	Min/+	13	54.2 ± 6.4 g	9.30 ± 0.24 h	0.63 ± 0.08 g
	db/m	Min/+	19	24.5 ± 1.8	9.13 ± 0.33	0.30 ± 0.03
	m/m	Min/+	10	20.8 ± 1.8	8.75 ± 0.23	0.27 ± 0.03

a Mean ± SD;

b Significantly different from the db/m-Min/+ and m/m-Min/+ males (*p* < 0.001);

c Significantly different from the m/m-Min/+ males (*p* < 0.05);

d Significantly different from the db/m-Min/+ females (*p* < 0.001);

e Significantly different from the db/m-Min/+ females (*p* < 0.01);

f Significantly different from the m/m-Min/+ females (*p* < 0.01);

g Significantly different from the db/m-Min/+ and m/m-Min/+ females (*p* < 0.001);

h Significantly different from the m/m-Min/+ females (*p* < 0.01).

**Table 2:** Serum levels of glucose, total cholesterol, triglyceride, and insulin of mice at 15 weeks of age.

Gender	Genotype		No. of mice examined	Glucose (mg/dL)	Total cholesterol (mg/dL)	Triglyceride (mg/dL)	Insulin (ng/dl)

	db/db type	Apc^Min/+^ type					
Males	db/db	Min/+	18	250.6 ± 35.6 a,b	161.8 ± 28.7 b	193.1 ± 14.8 c	3.3 ± 1.4 b
	db/m	Min/+	23	185.7 ± 27.6 d	109.4 ± 13.1	162.6 ± 27.6	0.4 ± 0.2
	m/m	Min/+	11	187.0 ± 21.8 e	96.1 ± 13.0	165.2 ± 21.5	0.3 ± 0.1

Females	db/db	Min/+	13	226.2 ± 28.7 f	145.1 ± 18.5 g	215.0 ± 38.0 h,i	3.5 ± 1.1 f
	db/m	Min/+	19	181.6 ± 24.5	119.1 ± 12.5	182.3 ± 29.8	0.3 ± 0.1
	m/m	Min/+	10	195.0 ± 20.4	115.3 ± 10.3	170.9 ± 18.2	0.3 ± 0.1

a Mean ± SD;

b Significantly different from the db/m-Min/+ and m/m-Min/+ males (*p* < 0.001);

c Significantly different from the db/m-Min/+ males (*p* < 0.01);

d Significantly different from the db/m-Min/+ females (*p* < 0.01);

e Significantly different from the m/m-Min/+ females (*p* < 0.01);

f Significantly different from the db/m-Min/+ and m/m-Min/+ females (*p* < 0.001);

g Significantly different from the m/m-Min/+ females (*p* < 0.01);

h Significantly different from the db/m-Min/+ females (*p* < 0.05);

i Significantly different from the m/m-Min/+ females (*p* < 0.01).

**Table 3:** Multiplicity (no. of tumors/mouse) and incidence of intestinal tumors (adenomas) of mice at 15 weeks of age.

Gender	Genotype		No. of mice examined	Total number of adenomas/mouse (incidence)	Small intestine (incidence)	Colon (incidence)

	db/db type	Apc^Min/+^ type				
Males	db/db	Min/+	18	60.5 ± 14.6 a,b (18/18, 100%)	58.8 ± 13.7 b (18/18, 100%)	1.7 ± 2.6 (12/18, 66.7% c)
	db/m	Min/+	23	32.1 ± 7.5 (23/23, 100%)	31.6 ± 6.7 (23/23, 100%)	0.6 ± 1.2 (5/23, 21.8%)
	m/m	Min/+	11	30.5 ± 7.7 (11/11, 100%)	29.5 ± 6.1 (11/11, 100%)	0.9 ± 2.1 (3/11, 27.3%)

Females	db/db	Min/+	13	57.8 ± 12.7 d (13/13, 100%)	58.6 ± 12.3 d (13/13, 100%)	1.2 ± 1.5 (7/13, 53.8%)
	db/m	Min/+	19	32.6 ± 5.9 (19/19, 100%)	32.4 ± 5.9 (19/19, 100%)	0.2 ± 0.4 (4/19, 21.1%)
	m/m	Min/+	10	30.9 ± 6.3 (10/10, 100%)	30.7 ± 6.3 (10/10, 100%)	0.2 ± 0.4 (2/10, 20.0%)

a Mean ± SD;

b Significantly different from the db/m-Min/+ and m/m-Min/+ males (*p* < 0.001);

c Significantly different from the db/m-Min/+ and m/m-Min/+ males (*p* < 0.005);

d Significantly different from the db/m-Min/+ and m/m-Min/+ females (*p* < 0.001).
