# Supplementary material for: C57BL/KsJ-db/db-ApcMin/+ Mice Exhibit an Increased Incidence of Intestinal Neoplasms
Source: Int J Mol Sci. 2011 Nov 18;12(11):8133–45. doi: 10.3390/ijms12118133 (PMC3233461; doi:10.3390/ijms12118133)
Supplement: Supplementary file 1 [file ijms-12-08133-s001.pdf]

Supplementary Information

Kazuya Hata [1,2,]*, Masaya Kubota [3], Masahito Shimizu [3], Hisataka Moriwaki [3], Toshiya Kuno [1], Takuji Tanaka [1,4,5,]*, Akira Hara [1] and Yoshinobu Hirose [1]

1 Department of Tumor Pathology, Gifu University Graduate School of Medicine, 1–1 Yanagido, Gifu 501–1194, Japan; E-Mails: tkuno@gifu-u.ac.jp (T.K.); ahara@gifu-u.ac.jp (A.H.); yhirose@gifu-u.ac.jp (Y.H.)

2 Kamiishidu Division, Sunplanet Co., Gifu 503–1602, Japan

3 Department of Medicine, Gifu University Graduate School of Medicine, Gifu 501–1194, Japan; E-Mails: samurai0201@yahoo.co.jp (M.K.); shimim-gif@umin.ac.jp (M.S.); hmori@gifu-u.ac.jp (H.M.)

4 Department of Oncologic Pathology, Kanazawa Medical University, Ishikawa 920–0293, Japan

5 Cancer Research and Prevention (TCI-CaRP), Tohkai Cytopathology Institute, Gify 500–8285, Japan

* Authors to whom correspondence should be addressed; E-Mails: k-hata-sun@hhc.eisai.co.jp (K.H.); takutt@toukaisaibou.co.jp (T.T.); Tel.: +81-584-46-3241 (K.H.); +81-58-273-4399 (T.T.); Fax: +81-584-48-001 (K.H.); +81-58-273-4392 (T.T.).

Received: 8 October 2011; in revised form: 30 October 2011 / Accepted: 11 November 2011 / Published: 18 November 2011

Abstract: The numbers of obese people and diabetic patients are ever increasing. Obesity and diabetes are high-risk conditions for chronic diseases, including certain types of cancer, such as colorectal cancer (CRC). The aim of this study was to develop a novel animal model in order to clarify the pathobiology of CRC development in obese and diabetic patients. We developed an animal model of obesity and colorectal cancer by breeding the C57BL/KsJ-*db/db* (db/db) mouse, an animal model of obesity and type II diabetes, and the C57BL/6J-$Apc^{Min/+}$ (Min/+) mouse, a model of familial adenomatous polyposis. At 15 weeks of age, the N9 backcross generation of C57BL/KsJ-*db/db*-$Apc^{Min/+}$ (db/db-Min/+) mice developed an increased incidence and multiplicity of adenomas in the intestinal tract when compared to the db/m-Min/+ and m/m-Min/+ mice. Blood biochemical profile showed significant increases in insulin (8.3-fold to 11.7-fold), cholesterol (1.2-fold to 1.7-fold), and triglyceride (1.2-fold to 1.3-fold) in the db/db-Min/+ mice, when compared to those of the db/m-Min/+ and m/m-Min/+ mice. Increases (1.4-fold to 2.6-fold) in RNA levels of insulin-like growth factor (IGF)-1, IRF-1R, and IGF-2 were also observed in the db/db-Min/+ mice. These results suggested that the IGFs, as well as hyperlipidemia and hyperinsulinemia, promoted adenoma formation in the db/db-Min/+ mice. Our results thus suggested that the db/db-Min/+ mice should be invaluable for studies on the pathogenesis of CRC in obese and diabetes patients and the therapy and prevention of CRC in these patients.

Keywords: C57BL/KsJ-db/db; C57BL/6J-$Apc^{Min/+}$; Type 2 diabetes mellitus; colon carcinogenesis; animal model

---

Figure S1. N9 backcross generation.

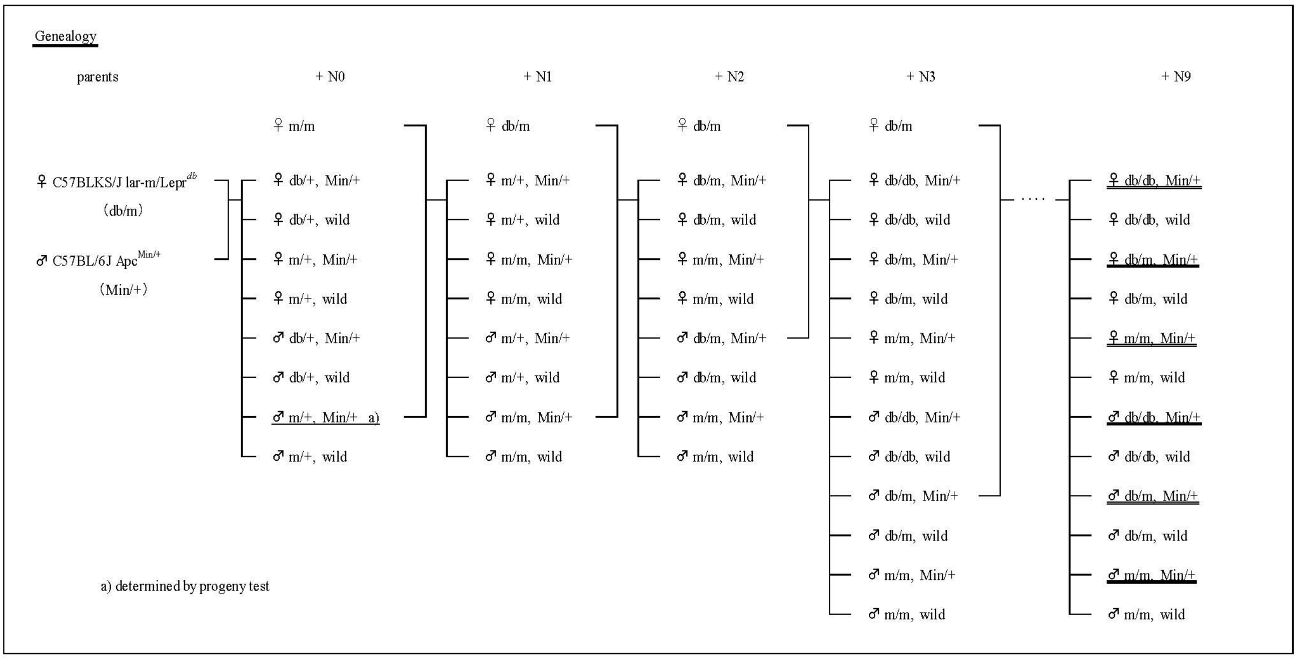

© 2011 by the authors; licensee MDPI, Basel, Switzerland. This article is an open access article distributed under the terms and conditions of the Creative Commons Attribution license (http://creativecommons.org/licenses/by/3.0/).
